# Epidemiology of community-onset *Staphylococcus aureus *infections in pediatric patients: an experience at a Children's Hospital in central Illinois

**DOI:** 10.1186/1471-2334-9-112

**Published:** 2009-07-16

**Authors:** Kanokporn Mongkolrattanothai, Jean C Aldag, Peggy Mankin, Barry M Gray

**Affiliations:** 1 Department of Pediatrics, Division of Pediatric Infectious Diseases, University of Illinois College of Medicine at Peoria and Children's Hospital of Illinois at OSF Saint Francis Medical Center, Illinois, USA; 2 Department of Internal Medicine, University of Illinois College of Medicine at Peoria, Illinois, USA

## Abstract

**Background:**

The nation-wide concern over methicillin-resistant *Staphylococcus aureus *(MRSA) has prompted many clinicians to use vancomycin when approaching patients with suspected staphylococcal infections. We sought to characterize the epidemiology of community-onset *S. aureus *infections in hospitalized children to assist local clinicians in providing appropriate empiric antimicrobial therapy.

**Methods:**

From January 2005–June 2008, children (0–18 years old) admitted to the Children's Hospital of Illinois with community-onset *S. aureus *infections were identified by a computer-assisted laboratory-based surveillance and medical record review.

**Results:**

Of 199 patients, 67 (34%) had invasive infections, and 132 (66%) had skin and soft tissue infections (SSTIs). Among patients with invasive infections, *S. aureus *isolates were more likely to be susceptible to methicillin (MSSA 63% vs. MRSA 37%), whereas patients with SSTIs, *S. aureus *isolates were more likely to be resistant to methicillin (MRSA 64% vs. MSSA 36%). Bacteremia and musculoskeletal infections were the most common invasive infections in both groups of *S. aureus*. Pneumonia with empyema was more likely to be caused by MRSA (*P *= 0.02). The majority (~90%) of MRSA isolates were non-multidrug resistant, even in the presence of healthcare-associated risk factors.

**Conclusion:**

Epidemiological data at the local level is important for antimicrobial decision-making. MSSA remains an important pathogen causing invasive community-onset *S. aureus *infections among hospitalized children. In our hospital, nafcillin in combination with vancomycin is recommended empiric therapy in critically ill patients with suspected invasive staphylococcal infections. Because up to 25% of MSSA circulating in our area are clindamycin-resistant, clindamycin should be used cautiously as empiric monotherapy in patients with suspected invasive staphylococcal infections.

## Background

Infections caused by community-associated methicillin-resistant *Staphylococcus aureus *(CA-MRSA) have been increasingly reported worldwide. Such isolates differ from healthcare-associated MRSA (HA-MRSA) by a distinct antimicrobial susceptibility pattern, usually being susceptible to non-β-lactam antimicrobial agents and having different genetic backgrounds as determined by SCC*mec *elements, multilocus sequence types (MLST), and pulsed-field gel electrophoresis [1-4]. Many studies suggest that CA-MRSA have replaced their methicillin-susceptible counterparts as the major cause of skin and soft tissue infections and other invasive diseases [5-9]. These findings have important clinical implications for the selection of antimicrobial agents. In areas where CA-MRSA is common, antimicrobial agents that are active against CA-MRSA should be advocated for empiric treatment of patients with potential *S. aureus *infections, until the culture and susceptibility results are available [5,8-10].

It is important to note that most data on CA-MRSA come from large tertiary care centers, many of which are located in large metropolitan areas. Thus, these data may not be generalizable to other hospitals in smaller, mid-size or more rural communities. Knowledge of the antimicrobial susceptibility patterns at the local level is essential for selecting appropriate empiric therapy of the wide variety of *S. aureus *infections. At the Children's Hospital of Illinois (CHOI), we have observed significant numbers of patients with community-onset infections caused not only by MRSA but also by MSSA. We thus needed a better understanding of the epidemiology of community-onset *S. aureus *infections in children admitted to our institution.

## Methods

### Setting

The Children's Hospital of Illinois at OSF Saint Francis Medical Center has 127 inpatient beds and about 5000 admissions annually. It serves as the academic, tertiary care referral center for the central region of Illinois.

### Study design

A database of laboratory records from the OSF System Laboratory was used to identify pediatric patients 18 years of age or younger who were hospitalized from January 1, 2005 through June 30, 2008 and had microbiologic specimens that yielded *S. aureus*. For each case of *S. aureus *isolated from clinical specimens, the relevant medical information was examined, including diagnosis, onset of infection, infection sites, demographics (age, gender), underlying illnesses, risk factors for healthcare-associated infections, and isolate antimicrobial susceptibility. The study was approved by the Peoria Institutional Review Board.

### Definitions

Patients with community-onset *S. aureus *infections were included if (a) the patients' isolates were recovered within 48 hours of admission, based on the criteria established by the Centers for Disease Control and Prevention (CDC, Atlanta) or if (b) their isolates were obtained after 48 hours of admission but patients had clinical evidence of diseases prior to admission.

A case of invasive infection was defined by 1 or more of the following conditions: bacteremia, endocarditis, pneumonia, lymphadenitis, septic arthritis, osteomyelitis, or another illness in which *S. aureus *was isolated from normally sterile body fluids. Infections involving the skin or soft tissue structures such as abscess or cellulitis were regarded as skin and soft tissue infections (SSTIs).

Patients were excluded if they had positive culture results but no signs of infection; or if a diagnosis of MRSA infection was made on the basis of positive MRSA screening cultures (nose, axilla, perineum, or rectum); or if the diagnosis of staphylococcal pneumonia was based solely on the isolation of *S. aureus *from a tracheal aspirate or sputum. Patients with orbital or otogenic infections were excluded if the *S. aureus *isolates were recovered from the swabs of the eye or ear drainage only.

For individuals with multiple hospital admissions for SSTIs during a single year, data were obtained from the first hospitalization. Risk factors for healthcare-associated infections included hospitalization or surgery in the preceding 12 months, the presence of an indwelling catheter or a percutaneous device, or frequent exposure to a healthcare facility related to an underlying condition. A hospital birth without any postnatal complications was not considered a risk factor.

### Antimicrobial susceptibility testing

An automated system (Vitek 2; bioMérieux) was used to determine the antimicrobial susceptibility profile of *S. aureus *isolates in accordance with the recommendations of the Clinical and Laboratory Standards Institute. For isolates that tested resistant to erythromycin but susceptible to clindamycin, a D-test was performed to detect inducible resistance to clindamycin. Among MRSA, multidrug resistance (MDR) was used and defined as resistance to three or more non-β-lactam antimicrobial agents (ciprofloxacin or levofloxacin, clindamycin, erythromycin, gentamicin, tetracycline, trimethoprim-sulfamethoxazole, rifampin, vancomycin).

### Statistical analyses

Differences in variables between groups were calculated by chi-square test or Fisher's exact test, as appropriate. *P *< 0.05 was considered statistically significant.

## Results

We identified 212 hospitalized pediatric patients whose *S. aureus *infections were considered community-onset. Thirteen patients were excluded: four patients had no clinical evidence of diseases and received no antibiotic therapy, including three with a positive blood culture and one who grew *S. aureus *from the gall bladder after elective cholecystectomy; nine had invasive diseases, but their infections could not be definitely proved to be caused by *S. aureus*, including three with orbital cellulitis, two with otomastoiditis, and one each with pneumonia, retropharyngeal abscess, possible staphylococcal scalded skin syndrome, and fasciitis of the chest wall.

**Figure 1:**
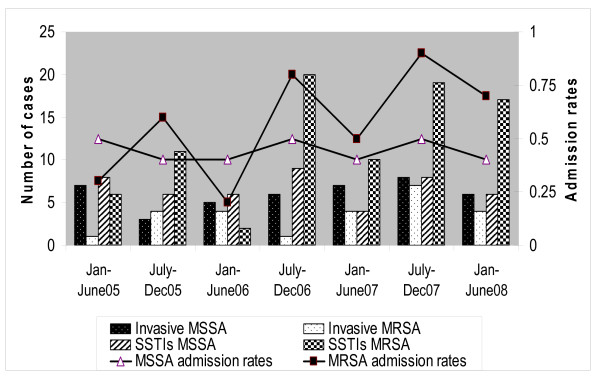
**Number of cases and admission rates (number of cases per 100 hospital admissions) of community-onset *S. aureus *infections among hospitalized pediatric patients during the study period**.

Of the remaining 199 patients, 67 (34%) had invasive infections and 132 (66%) had SSTIs. These two groups were significantly different (*P *< 0.01) with regard to the proportion of MSSA and MRSA isolates (Table 1). Among patients with invasive infections, *S. aureus *isolates were more likely to be susceptible to methicillin [MSSA 42/67 (63%) vs. MRSA 25/67 (37%)]. In patients with SSTIs, *S. aureus *isolates were more likely to be resistant to methicillin [MSSA 47/132 (36%) vs. MRSA 85/132 (64%)]. The semi-annual distribution of cases and admission rates (number of cases per 100 hospital admissions) are shown in Figure 1. Overall, the number of community-onset infections caused by MRSA increased yearly, but the increase was mainly due to SSTIs.

**Table 1:** Demographic and clinical features of patients with community-onset *S. aureus *infections.

	No. (%)		
		
Variables	MSSA n = 89	MRSA n = 110	P
Age			
0–3 mo	11 (12.4)	7 (6.4)	
4–59 mo	42 (47.2)	82 (74.5)	< 0.01
5–10 y	12 (13.5)	10 (9.1)	
11–18 y	24 (26.9)	11 (10.0)	
Gender			
Male	46 (51.7)	47 (42.7)	0.25
Female	43 (48.3)	63 (57.3)	
Clinical manifestations			
Skin and soft tissue infections	47 (52.8)	85 (77.3)	< 0.01
Invasive infections	42 (47.2)	25 (22.7)	
Healthcare-associated risk factors			
Present	31 (34.8)	33 (30.0)	0.57
Absent	58 (65.2)	77 (79.0)	

Demographic and clinical features of patients are summarized in Table 1. There was a significant age group difference (*P *< 0.01) between patients with MSSA and MRSA infections. MSSA infections were common in young children (4–59 mo) and teenagers (11–18 y), whereas MRSA infections occurred more often in young children (4–59 mo). Characteristics of invasive infections and healthcare-associated risk factors are shown in Table 2. Bacteremia and musculoskeletal infections were the most common invasive infections caused by *S. aureus*, regardless of its methicillin susceptibility. Fourteen patients (9 MSSA and 5 MRSA) had *S. aureus *bacteremia complicated with one or more site of infection. Among patients who had invasive infections, pneumonia with pleural empyema was more likely to be caused by MRSA (*P *= 0.02), although there were only 9 patients with pneumonia in this study. In addition, among patients with invasive infections, only 29 patients had a documented history whether or not they had prior MRSA skin infections or contact with MRSA infected persons or family members before the onset of the illness. Such history was found in 67% (12/18) of patients with invasive MRSA infections, and in only 9% (1/11) of patients with invasive MSSA infections (*P *< 0.01). The most commonly affected sites of SSTIs were perineum and buttocks (45%), especially in the 4–59 months age group.

**Table 2:** Characteristics of invasive infections and healthcare-associated risk factors.

	MSSA	MRSA
Invasive Infections		
Bacteremia	17	10
Cervical lymphadenitis	9	4
Endocarditis	2	1
Pneumonia	2	7
Musculoskeletal infections	11	7
Others	6^a^	4^b^
		
Healthcare-associated Risk factors		
Previous hospitalization	28	28
Previous surgery	20	15
Presence of an indwelling catheter or a percutaneous device	5	5
Underlying medical illnesses		
Cystic fibrosis	1	1
Congenital heart diseases	4	-
Diabetes	1	1
Immunosuppressive therapy	2	1
Hemophilia	1	-
Renal failure	-	1
Short-bowel syndrome	-	1

^a^Pericarditis (1), retropharyngeal abscess (1), staphylococcal toxic shock syndrome (1), chest wall abscess (1), suppurative parotitis (1), infection of the pace maker (1).

^b^Meningitis (1), dialysis catheter-related peritonitis (1), subhepatic abscess (1), infection of the implanted baclofen pump (1).

Exposure to healthcare-associated risk factors was not significantly different between MSSA and MRSA groups (*P *= 0.57). In the subgroup of patients who had no documented healthcare-associated risk factors, bacteremia (n = 9) and musculoskeletal infections (n = 9) remained the most common invasive infections caused by MSSA, whereas pneumonia with pleural empyema (n = 7) was the most common invasive infection caused by MRSA.

Clindamycin resistance (constitutive and inducible resistance) was observed in 15% (13 of 89) of MSSA. However, the proportion of MSSA resistant to clindamycin was 21% (10 of 47) among isolates causing SSTIs, but was not significantly different (7%, 3 of 42) among isolates causing invasive infections (*P *= 0.08). Antimicrobial susceptibility testings of MRSA isolates are shown in Additional file 1. The majority of MRSA isolates were non-MDR (88% vs. 93% in patients with and without healthcare-associated risk factors, respectively) (Table 3). Only 9 MRSA isolates were MDR, of which 4 were in patients who had healthcare-associated risk factors. There were no significant differences in healthcare-associated risk factors among patients who had infections caused by non-MDR (*P *= 0.20) or MDR (*P *= 0.21) MRSA. In our population, presence of risk factors for healthcare-associated infections was not associated with MDR MRSA infections (*P *= 0.45).

**Table 3:** Resistance patterns of MRSA isolates* stratified by healthcare-associated (HA) risk factors.

	No. (%)		
		
	Patients with HA risk factors	Patients without HA risk factors	*P*
Non-MDR MRSA isolates	n = 29	n = 70	0.20
Invasive infections	9 (9)	12 (12)	
SSTIs	20 (20)	58 (59)	
			
MDR MRSA isolates	n = 4	n = 5	0.21
Invasive infections	3 (33)	1 (11)	
SSTIs	1 (11)	4 (45)	

* MDR phenotype was not determined in 2 MRSA isolates that were susceptible to clindamycin but resistant to erythromycin and tetracycline because of lack of D test results. These 2 MRSA isolates caused skin infections in patients without healthcare-associated risk factors.

## Discussion

In this study, the percentages of *S. aureus *causing SSTIs that are methicillin-resistant have increased yearly and accounted for 70–75% during 2007–2008. This finding was not different from other regions of the United States. Nevertheless, MSSA remains a common pathogen causing invasive community-onset *S. aureus *infections. It is of note that, in year 2007, there may have been a trend toward an increase in invasive MRSA infections (Figure 1). However, in the nine months since the end of this study, we have seen 13 more cases of community-onset invasive MSSA infections, but only 2 cases of community-onset invasive MRSA infections. The finding that MSSA was the predominant pathogen of invasive staphylococcal infections might not be unexpected in a region of low MRSA prevalence such as western Sweden [11]. A recent study of community-associated *S. aureus *infections in Greece was similar to our findings that MSSA predominated among children with invasive infections, whereas CA-MRSA predominated among children with SSTIs [12]. McCaskill et al. reported the increase of invasive infections caused by MSSA, of which 35% of isolates were related to USA300, the predominant clone of CA-MRSA [13]. It is plausible that some USA300 strains may have lost their SCC*mec *elements, thereby becoming MSSA, or alternatively, the ancestor of CA-MRSA USA300 was from an MSSA clone that acquired its resistance before spreading in the community [10].

The majority of community-onset MRSA isolates from hospitalized children, including individuals with healthcare-associated risk factors, were non-MDR – a characteristic commonly observed in molecularly defined "CA-MRSA" isolates. In this study, presence of healthcare-associated risk factors was neither associated with multidrug-resistant MRSA infections nor reliably predictive of the susceptibility to methicillin in *S. aureus *isolates causing community-onset infections. The use of healthcare-associated risk factors in exclusion criteria in studies of CA-MRSA epidemiology may have underestimated the CA-MRSA burden. Conversely, infections in patients who have healthcare-associated risk factors are diverse and, therefore, they are at increased risk for *S. aureus *infection not only by MRSA, but by MSSA as well.

The emergence of MRSA infections in the community has prompted clinicians to prescribe antibiotics that are active against CA-MRSA in patients with any suspected *S. aureus *infection. At CHOI, we have observed a significant increase in the use of vancomycin as empiric therapy, driven by growing concern for CA-MRSA. This practice has contributed to inappropriate vancomycin use and has been detrimental in several cases of invasive MSSA infections. A number of studies have demonstrated that vancomycin is inferior to β-lactams for the treatment of invasive MSSA infections [14-16]. Higher infection-related mortality has been reported in patients with invasive MSSA infections, even if vancomycin was switched to β-lactams once the culture results became available [14]. Clindamycin, another antibiotic that is active against many CA-MRSA and MSSA isolates, has been used empirically [17]. However, based on the surveillance for antimicrobial resistance among *S. aureus *circulating in our area (data not shown), the percentage of clindamycin resistance in MSSA isolates from 2005 to 2008 has been 20–25%. Because invasive *S. aureus *infections are associated with increased morbidity and mortality, and MSSA remains the predominant cause of invasive *S. aureus *infections, it is prudent for us to administer nafcillin in combination with vancomycin in critically ill patients with invasive staphylococcal infections, until susceptibility results are known. This recommendation has been supported and discussed elsewhere [18,19].

In contrast, the majority of community-onset MRSA isolates were non-MDR and were susceptible to clindamycin. Clindamycin is the recommended empirical anti-staphylococcal therapy for hospitalized pediatric patients with clinical syndromes likely caused by CA-MRSA, such as cutaneous abscesses or pneumonia with empyema. Its use avoids unnecessary exposure to vancomycin. In addition, the penetration of vancomycin into lung tissue and pulmonary lining fluid has been reported to be relatively low [20,21] and may limit the effectiveness of vancomycin in the therapy of MRSA pneumonia. Moreover, clindamycin inhibits toxin synthesis [22,23], including Panton-Valentine leukocidin, a toxin which is associated with suppurative skin and soft tissue infections and necrotizing pneumonia [24-26]. It also may be responsible for the increased virulence in some CA-MRSA isolates [1,27]. However, clindamycin is not appropriate for the treatment of endocarditis because of a high rate of relapse, presumed to be due to its bacteriostatic action, nor is it appropriate for meningitis because of poor central nervous system penetration. It should not be used as the empiric monotherapy in critically ill patients. There is also a risk of treatment failure during therapy if the isolate exhibits inducible clindamycin resistance [28]. At CHOI, to minimize the development of antimicrobial resistance, the empiric use of linezolid or daptomycin is discouraged. Once the minimal inhibitory concentrations (MICs) are available, for MRSA isolates in which vancomycin MICs are 1.5–2 mg/L it would be appropriate to treat MRSA infections with linezolid or daptomycin, since failure rate is higher for such organisms treated with vancomycin.

There are limitations to our study. Strict inclusion and exclusion criteria were used, thus possibly underestimating the true prevalence of community-onset *S. aureus *infections among hospitalized children. Information about other potentially important risk factors was not always documented in medical records, particularly the history of MRSA skin infections among patients or family members. Nevertheless, the findings from our study have clinically relevant implications for patient management. While it is important that clinicians be aware of the emergence of CA-MRSA infections, the diversity of epidemiology of *S. aureus *infections should also be recognized. Because antimicrobial resistance continues to evolve, it is imperative to continue monitoring *S. aureus *infections at the local level. This provides valuable data on resistance trends and contributes to more effective treatment recommendations for local and regional use.

## Conclusion

We have described the characteristics of community-onset *S. aureus *infections among hospitalized children in Central Illinois. We found that MRSA is increasing as a cause of skin and soft tissue infections, but that MSSA remains a common cause of invasive infections. In our institution, nafcillin in combination with vancomycin is recommended empiric therapy in critically ill patients with suspected invasive staphylococcal infections. Because up to 25% of MSSA circulating in our area are clindamycin-resistant, clindamycin should be used cautiously as empiric monotherapy in patients with suspected invasive staphylococcal infections unless antimicrobial susceptibility is known.

## Competing interests

The authors declare that they have no competing interests.

## Authors' contributions

KM and BG conceived and designed the study. KM wrote the first draft of the paper and other coauthors contributed to the final draft. KM was responsible for conducting the study and managing the data. KM and JCA conducted the statistical analyses and the interpretation of data. Others participated in data analysis and data interpretation. All authors read and approved the final manuscript.

## Pre-publication history

The pre-publication history for this paper can be accessed here:

http://www.biomedcentral.com/1471-2334/9/112/prepub

## Supplementary Material

Additional file 1 **Antimicrobial susceptibility pattern of MRSA isolates stratified by type of infections and healthcare-associated risk factors**. The data provided represent antimicrobial susceptibility patterns of MRSA isolates stratified by type of infections and healthcare-associated risk factors. Click here for file

## References

[B1] Boyle-Vavra S Daum RS Community-acquired methicillin-resistant *Staphylococcus aureus*: the role of Panton-Valentine leukocidin Lab Invest 2007 87 3 9 10.1038/labinvest.3700501 17146447

[B2] Fey PD Said-Salim B Rupp ME Hinrichs SH Boxrud DJ Davis CC Kreiswirth BN Schlievert PM Comparative molecular analysis of community- or hospital-acquired methicillin-resistant *Staphylococcus aureus* Antimicrob Agents Chemother 2003 47 196 203 12499191 10.1128/AAC.47.1.196-203.2003 PMC149027

[B3] Naimi TS LeDell KH Como-Sabetti K Borchardt SM Boxrud DJ Etienne J Johnson SK Vandenesch F Fridkin S O'Boyle C Danila RN Lynfield R Comparison of community- and health care-associated methicillin-resistant *Staphylococcus aureus *infection JAMA 2003 290 2976 2984 10.1001/jama.290.22.2976 14665659

[B4] Okuma K Iwakawa K Turnidge JD Grubb WB Bell JM O'Brien FG Coombs GW Pearman JW Tenover FC Kapi M Tiensasitorn C Ito T Hiramatsu K Dissemination of new methicillin-resistant *Staphylococcus aureus *clones in the community J Clin Microbiol 2002 40 4289 4294 12409412 10.1128/JCM.40.11.4289-4294.2002 PMC139674

[B5] Moran GJ Krishnadasan A Gorwitz RJ Fosheim GE McDougal LK Carey RB Talan DA Methicillin-resistant *S. aureus *infections among patients in the emergency department N Engl J Med 2006 355 666 674 10.1056/NEJMoa055356 16914702

[B6] Frazee BW Lynn J Charlebois ED Lambert L Lowery D Perdreau-Remington F High prevalence of methicillin-resistant *Staphylococcus aureus *in emergency department skin and soft tissue infections Ann Emerg Med 2005 45 311 320 10.1016/j.annemergmed.2004.10.011 15726056

[B7] Gonzalez BE Hulten KG Dishop MK Lamberth LB Hammerman WA Mason EO Jr Kaplan SL Pulmonary manifestations in children with invasive community-acquired *Staphylococcus aureus *infection Clin Infect Dis 2005 41 583 590 10.1086/432475 16080077

[B8] Pannaraj PS Hulten KG Gonzalez BE Mason EO Jr Kaplan SL Infective pyomyositis and myositis in children in the era of community-acquired, methicillin-resistant *Staphylococcus aureus *infection Clin Infect Dis 2006 43 953 960 10.1086/507637 16983604

[B9] Baker C Large CA-MRSA disease burden mandates prompt diagnosis, appropriate management AAP News 2007 28 1 and 9

[B10] Miller LG Perdreau-Remington F Bayer AS Diep B Tan N Bharadwa K Tsui J Perlroth J Shay A Tagudar G Ibebuogu U Spellberg B Clinical and epidemiologic characteristics cannot distinguish community-associated methicillin-resistant *Staphylococcus aureus *infection from methicillin-susceptible *S. aureus *infection: a prospective investigation Clin Infect Dis 2007 44 471 482 10.1086/511033 17243048

[B11] Jacobsson G Dashti S Wahlberg T Andersson R The epidemiology of and risk factors for invasive *Staphylococcus aureus *infections in western Sweden Scand J Infect Dis 2007 39 6 13 10.1080/00365540600810026 17366006

[B12] Sdougkos G Chini V Papanastasiou DA Christodoulou G Stamatakis E Vris A Christodoulidi I Protopapadakis G Spiliopoulou I Community-associated *Staphylococcus aureus *infections and nasal carriage among children: molecular microbial data and clinical characteristics Clin Microbiol Infect 2008 14 995 1001 10.1111/j.1469-0691.2008.02064.x 18808423

[B13] McCaskill ML Mason EO Jr Kaplan SL Hammerman W Lamberth LB Hulten KG Increase of the USA300 clone among community-acquired methicillin-susceptible *Staphylococcus aureus *causing invasive infections Pediatr Infect Dis J 2007 26 1122 1127 10.1097/INF.0b013e31814536e0 18043449

[B14] Lodise TP Jr McKinnon PS Levine DP Rybak MJ Impact of empirical-therapy selection on outcomes of intravenous drug users with infective endocarditis caused by methicillin-susceptible *Staphylococcus aureus* Antimicrob Agents Chemother 2007 51 3731 3733 17664322 10.1128/AAC.00101-07 PMC2043293

[B15] Chang FY Peacock JE Jr Musher DM Triplett P MacDonald BB Mylotte JM O'Donnell A Wagener MM Yu VL *Staphylococcus aureus *bacteremia: recurrence and the impact of antibiotic treatment in a prospective multicenter study Medicine (Baltimore) 2003 82 333 339 10.1097/01.md.0000091184.93122.09 14530782

[B16] Kim SH Kim KH Kim HB Kim NJ Kim EC Oh MD Choe KW Outcome of vancomycin treatment in patients with methicillin-susceptible *Staphylococcus aureus *bacteremia Antimicrob Agents Chemother 2008 52 192 197 17984229 10.1128/AAC.00700-07 PMC2223910

[B17] Martinez-Aguilar G Hammerman WA Mason EO Jr Kaplan SL Clindamycin treatment of invasive infections caused by community-acquired, methicillin-resistant and methicillin-susceptible *Staphylococcus aureus *in children Pediatr Infect Dis J 2003 22 593 598 10.1097/00006454-200307000-00006 12867833

[B18] Kaplan SL Community-acquired methicillin-resistant *Staphylococcus aureus *infections in children Semin Pediatr Infect Dis 2006 17 113 119 10.1053/j.spid.2006.06.004 16934705

[B19] Baker C Large CA-MRSA disease burden mandates prompt diagnosis, appropriate management AAP News 2007 28 1 9

[B20] Cruciani M Gatti G Lazzarini L Furlan G Broccali G Malena M Franchini C Concia E Penetration of vancomycin into human lung tissue J Antimicrob Chemother 1996 38 865 869 10.1093/jac/38.5.865 8961057

[B21] Lamer C de Beco V Soler P Calvat S Fagon JY Dombret MC Farinotti R Chastre J Gibert C Analysis of vancomycin entry into pulmonary lining fluid by bronchoalveolar lavage in critically ill patients Antimicrob Agents Chemother 1993 37 281 286 8452359 10.1128/aac.37.2.281 PMC187653

[B22] Dumitrescu O Boisset S Badiou C Bes M Benito Y Reverdy ME Vandenesch F Etienne J Lina G Effect of antibiotics on *Staphylococcus aureus *producing Panton-Valentine leukocidin Antimicrob Agents Chemother 2007 51 1515 1519 17242137 10.1128/AAC.01201-06 PMC1855455

[B23] Stevens DL Ma Y Salmi DB McIndoo E Wallace RJ Bryant AE Impact of antibiotics on expression of virulence-associated exotoxin genes in methicillin-sensitive and methicillin-resistant *Staphylococcus aureus* J Infect Dis 2007 195 202 211 10.1086/510396 17191165

[B24] Gillet Y Issartel B Vanhems P Fournet JC Lina G Bes M Vandenesch F Piemont Y Brousse N Floret D Etienne J Association between *Staphylococcus aureus *strains carrying gene for Panton-Valentine leukocidin and highly lethal necrotising pneumonia in young immunocompetent patients Lancet 2002 359 753 759 10.1016/S0140-6736(02)07877-7 11888586

[B25] Yamasaki O Kaneko J Morizane S Akiyama H Arata J Narita S Chiba J Kamio Y Iwatsuki K The association between *Staphylococcus aureus *strains carrying panton-valentine leukocidin genes and the development of deep-seated follicular infection Clin Infect Dis 2005 40 381 385 10.1086/427290 15668860

[B26] Lina G Piemont Y Godail-Gamot F Bes M Peter MO Gauduchon V Vandenesch F Etienne J Involvement of Panton-Valentine leukocidin-producing *Staphylococcus aureus *in primary skin infections and pneumonia Clin Infect Dis 1999 29 1128 1132 10.1086/313461 10524952

[B27] Bocchini CE Hulten KG Mason EO Jr Gonzalez BE Hammerman WA Kaplan SL Panton-Valentine leukocidin genes are associated with enhanced inflammatory response and local disease in acute hematogenous *Staphylococcus aureus *osteomyelitis in children Pediatrics 2006 117 433 440 10.1542/peds.2005-0566 16452363

[B28] Siberry GK Tekle T Carroll K Dick J Failure of clindamycin treatment of methicillin-resistant *Staphylococcus aureus *expressing inducible clindamycin resistance in vitro Clin Infect Dis 2003 37 1257 1260 10.1086/377501 14557972

