# Supplementary material for: Epidemiology of community-onset Staphylococcus aureus infections in pediatric patients: an experience at a Children's Hospital in central Illinois
Source: BMC Infect Dis. 2009 Jul 16;9:112. doi: 10.1186/1471-2334-9-112 (PMC2722661; doi:10.1186/1471-2334-9-112)
Supplement: Additional file 1 — Antimicrobial susceptibility pattern of MRSA isolates stratified by type of infections and healthcare-associated risk factors. The data provided represent antimicrobial susceptibility patterns of MRSA isolates stratified by type of infections and healthcare-associated risk factors. [file 1471-2334-9-112-S1.doc]

Table 4. Antimicrobial susceptibility pattern of all MRSA isolates stratified by category of infection and healthcare-associated (HA) risk factors. Values are percentages of MRSA isolates that are susceptible to each antimicrobial agent.

|  | Ery | Clin | Cipro/ Levo | Gen | Tet | TMP- SMX | Rif | Vanc |
| --- | --- | --- | --- | --- | --- | --- | --- | --- |
| **SSTIs** |  |  |  |  |  |  |  |  |
| Patients with   HA risk factors  (n = 21) | 14 | 95 | 80 | 95 | 100 | 100 | 100 | 100 |
| Patients without  HA risk factors  (n = 64) | 8 | 94 | 94 | 100 | 92 | 100 | 100 | 100 |
| **Invasive infections** |  |  |  |  |  |  |  |  |
| Patients with   HA risk factors  (n =12) | 0 | 58 | 64 | 100 | 92 | 100 | 100 | 100 |
| Patients without  HA risk factors  (n = 13) | 8 | 85 | 92 | 100 | 100 | 100 | 100 | 100 |

Ery, erythromycin; Clin, clindamycin; Cipro/Levo, ciprofloxacin or levofloxacin; Gen, gentamicin; Tet, tetracycline; TMP-SMX, trimethoprim-sulfamethoxazole; Rif, rifampin; Vanc, vancomycin.
